# The Nurse's Part in Nursing Reforms

**Published:** 1918-11-23

**Authors:** 


### THE MATRONS' AND SISTERS' DEPARTMENT.

### THE NURSE'S PART IN NURSING REFORMS.

We have laid stress on the part taken by training-schools and superintendents of nurses in advancing the status of nurses and securing better conditions of work, but it is not to be supposed for a moment that matrons can be dissociated from the rest of the nursing staff in these establishments. By virtue of their office they have more power in their hands. But their aspirations do not differ from those of the nurses under them, except in so far as they have become more clearly defined in the course of the varied experience which has landed them in their present posts. It is never sufficiently borne in mind that matrons form no separate caste as do officers in the Navy and Army. They have served their apprenticeship to their business precisely in the same way as the lowliest little probationer who trembles at their appearance on the scene. Moreover, the ideals which inspire them in office had no sudden birth. They were stirring long before it seemed in the least probable that power would ever be lent to bring them to realities. The ordinary training-school is full of potential matrons. It is in the pupil stage, as she slowly progresses through various departments in her own and other training-schools, that the nurse acquires conviction of defects in the system. She knows what is wrong, not through the medium of rhetoric nor yet through second-hand stories coloured by the narrator's temperament, but by wearing the shoe herself and bearing the ache of the pinch.

The nurse of true reforming type does not stop short at recognition of what is wrong. Ceaselessly she tries to form a notion in her own mind as to how the evils from which she suffers can be abolished. As staff nurse she sets to work to make the lot of the probationers who work with her a little less difficult. As sister she already begins to make plans in consultation with her matron to lessen the strain for the little family under her and make her wards a place of harmonious co-ordination. As home sister, night superintendent, assistant matron, she gets increased scope for testing her theories and learning the limitations imposed on zeal. At last comes her opportunity as matron in some small or special hospital of introducing real innovations. She now learns the additional lesson that she cannot move without the concurrence of her colleagues and superior officers. She discovers that the talent of getting others to move in step along the rugged path of reform is the most difficult, yet by far the most valuable, which the reformer can cultivate. She practises that guileless deceit, dear to the true reformer, of instilling her own ideas into the powers that be while apparently receiving words of wisdom meekly from their lips. In short, she cares little who has the credit if she can but get things done. This is the manner in which nurses passing into the matron stage bring about advances in their profession. At every step their theories are severely tested. Alas! in too many cases it must be admitted that the springs of energy and progress become blunted before the time for action arrives.

Let it be supposed that the nurse with ideas should elect to take up private nursing instead of a hospital career. Is it not evident that so far as influencing the lot of her sisters is concerned she has hitherto been lost to the nursing world? Her influence may have been more widespread than that of the hospital nurse in other directions, but she could not influence the lives of her fellow-nurses except indirectly. The College of Nursing, with its network of Centres, is restoring her a place in the world of action. She can associate herself with the work for nurses carried on by those who direct nursing education. She can swell the majority which helps to carry forward the great movement, even though by herself she is powerless. And the same is true of other branches of work, although as a municipal or district nurse she may travel along a road similar to that we have pictured in the case of the hospital nurse. She may rise from one position of authority to another, gathering influence and experience all the time, until she reaches a post in which the fruits of energy, self-restraint, and open-mindedness are recognised by her fellow-workers and she stands confessed a leader of nursing opinion.

The intense isolation in which the nurse's life has hitherto been passed, in all its stages, private nursing, institution, or public Service, is slowly melting away. No longer are the pioneers doomed to clear a pathway through manifold obstructions, only to see the road closing once more behind them. No longer are those whose temperament does not incline them to the burdens of command compelled to drift in a back-water far from the current of nursing opinion. The College of Nursing is opening a sphere for all, within which their influence can make itself felt.

